# Clinical Biomarkers of Tumour Radiosensitivity and Predicting Benefit from Radiotherapy: A Systematic Review

**DOI:** 10.3390/cancers16101942

**Published:** 2024-05-20

**Authors:** Christopher W. Bleaney, Hebatalla Abdelaal, Mark Reardon, Carmel Anandadas, Peter Hoskin, Ananya Choudhury, Laura Forker

**Affiliations:** 1 Translational Radiobiology Group, Division of Cancer Sciences, The Oglesby Cancer Research Building, The University of Manchester, 555 Wilmslow Road, Manchester M20 4GJ, UKlaurajane.forker@nhs.net (L.F.); 2 Department of Clinical Oncology, The Christie NHS Foundation Trust, 550 Wilmslow Road, Manchester M20 4BX, UK

**Keywords:** biomarker, radiotherapy, radiosensitivity, gene expression signature

## Abstract

**Simple Summary:**

There are currently limited means by which radiotherapy treatment can be tailored to individual patient needs based on their tumour biology. Published studies of biomarkers aiming to predict tumour sensitivity to radiotherapy treatment for the clinic were reviewed. No gene expression signature biomarkers of sensitivity to radiotherapy are in routine clinical use, have been tested in advanced trials or are recommended for use in clinical guidelines. A pathway for future biomarker development that would enable tools for personalised clinical decision-making is proposed. This focusses on the need to improve the biological understanding of variation in sensitivity to radiotherapy, develop cost-effective assays and conduct large radiotherapy randomised controlled trials.

**Abstract:**

Modern advanced radiotherapy techniques have improved the precision and accuracy of radiotherapy delivery, with resulting plans being highly personalised based on individual anatomy. Adaptation for individual tumour biology remains elusive. There is an unmet need for biomarkers of intrinsic radiosensitivity that can predict tumour response to radiation to facilitate individualised decision-making, dosing and treatment planning. Over the last few decades, the use of high throughput molecular biology technologies has led to an explosion of newly discovered cancer biomarkers. Gene expression signatures are now used routinely in clinic to aid decision-making regarding adjuvant systemic therapy. They have great potential as radiotherapy biomarkers. A previous systematic review published in 2015 reported only five studies of signatures evaluated for their ability to predict radiotherapy benefits in clinical cohorts. This updated systematic review encompasses the expanded number of studies reported in the last decade. An additional 27 studies were identified. In total, 22 distinct signatures were recognised (5 pre-2015, 17 post-2015). Seventeen signatures were ‘radiosensitivity’ signatures and five were breast cancer prognostic signatures aiming to identify patients at an increased risk of local recurrence and therefore were more likely to benefit from adjuvant radiation. Most signatures (15/22) had not progressed beyond the discovery phase of development, with no suitable validated clinical-grade assay for application. Very few signatures (4/17 ‘radiosensitivity’ signatures) had undergone any laboratory-based biological validation of their ability to predict tumour radiosensitivity. No signatures have been assessed prospectively in a phase III biomarker-led trial to date and none are recommended for routine use in clinical guidelines. A phase III prospective evaluation is ongoing for two breast cancer prognostic signatures. The most promising radiosensitivity signature remains the radiosensitivity index (RSI), which is used to calculate a genomic adjusted radiation dose (GARD). There is an ongoing phase II prospective biomarker-led study of RSI/GARD in triple negative breast cancer. The results of these trials are eagerly anticipated over the coming years. Future work in this area should focus on (1) robust biological validation; (2) building biobanks alongside large radiotherapy randomised controlled trials with dose variance (to demonstrate an interaction between radiosensitivity signature and dose); (3) a validation of clinical-grade cost-effective assays that are deliverable within current healthcare infrastructure; and (4) an integration with biomarkers of other determinants of radiation response.

## 1. Introduction

The global burden of cancer is increasing, with 18.1 million new cases and 9.9 million cancer deaths reported in 2020. Cancers of the breast (12.5%), lung (12.2%), prostate (7.8%), rectum (4.0%), cervix (3.3%) and oesophagus (3.3%) are amongst the top ten new cancer diagnoses worldwide [1]. Radiotherapy is an integral component of curative treatment pathways for these cancers. It is well recognised that half of all cancer patients require radiotherapy at some point during the course of their treatment [2].

Modern techniques including image-guided radiotherapy (IGRT), magnetic resonance-guided adaptive radiotherapy (MRgART) and proton beam therapy (PBT) have improved the precision and accuracy of radiotherapy delivery, with resulting radiotherapy plans being highly personalised based on individual anatomy [3]. However, the response to radiotherapy is also dependent on tumour biology [4]. To adapt radiotherapy for biological factors, they must be reliably measurable in clinic. There is an important unmet need for validated biomarkers that can predict how a patient will respond to radiation and adapt their clinical management accordingly. This could facilitate decision-making between radical radiotherapy versus surgery or identify the need for dose escalation or radiotherapy-drug combinations for patients with less radioresponsive tumours.

The term ‘intrinsic radiosensitivity’ refers to inherent differences in individual tumour cell types in their sensitivity to radiation, which is independent of other biological factors such as hypoxia or proliferation [5]. The complexity and heterogeneity of genetic factors and pathways determining a tumour’s intrinsic radiosensitivity make this difficult to model. Therefore, there are fewer intrinsic radiosensitivity markers available than for other factors determining radiation response and none used as the standard of care.

The surviving fraction after exposure to 2 Gy ionising radiation (SF_2_) calculated using clonogenic assay is the gold standard measure of cellular intrinsic radiosensitivity in vitro [6]. This can be determined in pre-treatment ex vivo tumour specimens and correlates with clinical outcomes [7] but is not practical for clinical application. Early attempts to develop a surrogate biomarker to measure intrinsic radiosensitivity largely explored molecules in pathways involved in the response to DNA damage induced by ionising radiation. However, single molecule markers (often proteins) are vulnerable to intra-tumour heterogeneity and the reproducibility of assays across laboratories can be problematic [8].

The ‘omics’ era has led to an explosion in newly identified biomarkers over the last decade. Gene expression signatures have had great success in breast cancer treatment and there is now high-level evidence for their use in decision-making regarding adjuvant systemic therapy [9]. Assays are automated, reproducible, and not subject to inter-observer variation. Their potential as radiotherapy biomarkers is strengthened by the inclusion of multiple genes, making them more robust to intra-tumour heterogeneity than gene single markers [10]. This update to a previously published systematic review [11] is focused on gene expression signatures that have been evaluated in clinic for their ability to predict radiotherapy benefit. This work describes the recent large expansion in proposed signatures and evaluates their use in clinical decision-making.

## 2. Methods

### 2.1. Literature Search

This is an update to a previously published systematic review (Forker et al., 2015) [11]; therefore, searches were limited to 5 January 2015–1 January 2024.

A literature search of PubMed using the terms (predict OR prediction OR predictive OR predictor OR predicts) AND (radiotherapy OR radiosensitivity OR chemoradiotherapy) AND (‘radiosensitivity index’ OR gene signature OR molecular signature OR gene expression profile) was performed.

Searches were supplemented by the hand searching of reference lists of relevant studies and reviews. Abstracts were reviewed for all relevant titles and full papers obtained if necessary. This review was performed in accordance with the PRISMA (Preferred Reporting Items for Systematic Reviews and Meta-Analyses) guidelines and has not been registered.

### 2.2. Exclusion Criteria

Exclusion criteria are summarised in Table 1.

### 2.3. Definitions

Predictive value of radiotherapy benefit was defined as the differential association of the signature result with one or more clinical outcome measures between radiotherapy-treated patients and non-radiotherapy-treated patients with the same disease type. Assays were considered feasible for clinical implementation if the platform/sample type used to determine gene expression levels was in use in a phase III biomarker-led trial or in routine clinical practice and if the process used to assign a result can be applied prospectively (i.e., a whole cohort is not needed).

### 2.4. Gene Lists

Gene lists were obtained for each identified signature. Overlapping genes common to multiple signatures were identified and the proposed function of encoded proteins was obtained from UniProtKB/Swiss-Prot [12].

## 3. Results

**Figure 1:**
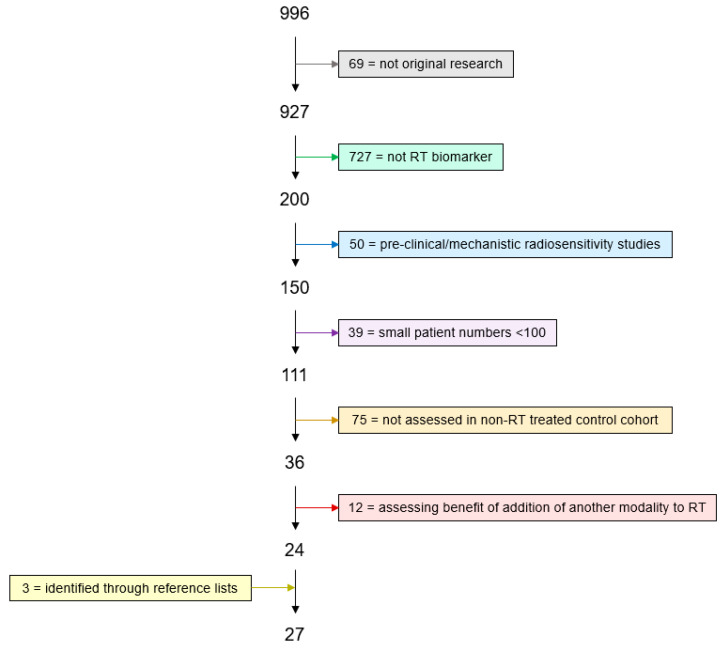
Literature search flow diagram. Citations identified and numbers excluded according to criteria in Table 1.

The literature search generated 996 citations plus 3 additional papers identified through reference lists. A flow diagram and reasons for exclusion are summarised in Figure 1. A total of 27 studies [13,14,15,16,17,18,19,20,21,22,23,24,25,26,27,28,29,30,31,32,33,34,35,36,37,38,39] including 19 signatures were found (Table 2). The previous systematic review covering up to 5 January 2015 yielded five studies [40,41,42,43,44] of five signatures (Table 3). The 32 studies from both searches included nine tumour types; the most common were breast (14 studies), glioma (5 studies) and head and neck squamous cell carcinoma (HNSCC) (3 studies).

Twenty-two distinct signatures (3–185 genes per signature) evaluated for their ability to predict radiotherapy benefit in clinical cohorts were identified in total. Seventeen signatures were described as ‘radiosensitivity’ signatures and five are prognostic breast cancer signatures. The 22 signatures contained 599 unique genes; 35 genes appeared in 2 signatures and 2 genes appeared in 4 signatures (Table 4).

Three radiosensitivity signatures were derived in vitro using whole transcriptome data from cell lines with known SF_2_ values and validated in clinical datasets for prognostic and predictive value. The remaining fourteen signatures were identified through bioinformatic studies using in vivo data from patients treated with radiotherapy. Genes were selected based on their ability to predict the outcome in patients treated with radiotherapy.

Three signatures were known as commercially available prognostic breast cancer signatures (Oncotype Dx/Oncotype DCIS, MammaPrint and Prosigna PAM-50) and two signatures were new prognostic breast cancer signatures designed specifically to identify patients likely to benefit from adjuvant radiotherapy.

### 3.1. Radiosensitivity Signatures Developed In Vitro

#### 3.1.1. Radiosensitivity Index (RSI) and Genomic-Adjusted Radiation Dose (GARD)

Torres-Roca et al. first proposed that gene expression could be used to predict radiosensitivity in vitro (SF_2_ measured using clonogenic assay) in 2005 [45]. The radiosensitivity index (RSI) is a score representing SF_2_ that is calculated from expression levels of ten genes using a rank-based linear algorithm. Forty-eight cell lines representing nine cancer types from the National Cancer Institute 60 (NCI-60) panel were used to develop the model, which was then shown to correctly predict SF_2_ in five of the nine remaining solid cancer cell lines [46]. The initial clinical evaluation demonstrated lower RSI in rectal cancer (n = 14) and oesophageal cancer (n = 12) patients responding to pre-operative chemoradiotherapy and an association of lower RSI with improved 2-year loco-regional control in HNSCC patients (n = 92) treated with radiotherapy and concurrent cisplatin-based chemotherapy [47].

The search criteria identified five studies in which RSI demonstrated predictive value in cohorts with n ≥ 100 patients including patients treated both with and without radiotherapy [prostate cancer [13] (n = 618), breast cancer [41] (two cohorts, n = 503), breast cancer [22] (n = 307), endometrial cancer [27] (n = 204), pancreatic ductal adenocarcinoma [30] (n = 589, prediction assessed in n = 56)]. A further study was included in which all patients were treated with radiotherapy for localised prostate cancer (n = 386) treated at a single institution, but with some patients receiving a relatively high dose with a high-dose rate (HDR) brachytherapy boost. RSI was able to identify patients more likely to benefit from higher dose radiotherapy [29].

More recently, it has been proposed that RSI can be integrated with the linear quadratic model, which is used clinically to estimate the biological effect of different dose-fractionation schedules. RSI is simply substituted for SF_2_ in the linear quadratic model of survival after d = 1 doses of n = 2 Gy to generate a tumour-specific α for an individual patient. This can then be used in the effect equation with the intended dose and fractionation to calculate a genomic-adjusted radiation dose (GARD). For a given radiotherapy dose, GARD will be higher for radiosensitive versus radioresistant tumours [48]. GARD was tested in a pooled, pan-cancer analysis of 11 previously published cohorts (seven cancer types) of 1615 patients treated with and without radiotherapy. GARD as a continuous variable was associated with overall survival (OS) and an interaction test demonstrates that this was dependent on whether a patient was treated with radiotherapy. An actual prescribed dose of radiation was not associated with clinical outcomes [49].

#### 3.1.2. 31-Gene Radiosensitivity Signature

The 31-gene radiosensitivity signature was also developed using the NCI-60 panel of cell lines and published data regarding their associated SF_2_ values were measured using clonogenic assay. Whole transcriptome data were obtained from four publicly available microarray studies and a linear regression model was used to identify genes whose expression correlated with SF_2_. A total of 31 genes were common to all microarray platforms and formed the final signature [50]. The first reported clinical evaluation was identified using the original literature search. This was a study of two cohorts of patients with primary brain tumours [Glioma—GSE16011 (n = 276, 193 received radiotherapy); Glioblastoma—the Cancer Genome Atlas (TCGA) (n = 463, all received radiotherapy)] [44]. The result was assigned as radiosensitive or radioresistant using hierarchical cluster analysis based on the expression of the 31 genes. Patients with radioresistant tumours had worse OS in radiotherapy-treated and non-radiotherapy-treated groups for GSE16011 on univariate analysis. This did not remain significant in the non-radiotherapy group after adjusting for age and performance status. The 31-gene signature was an independent predictor of OS in the TCGA cohort.

This updated systematic review revealed a further four studies of the 31-gene signature. For all the studies, the result was assigned using a cluster-based analysis of whole transcriptome data for entire cohorts. An HNSCC study included 288 patients from the TCGA (n = 214 radiotherapy, n = 74 no radiotherapy) and demonstrated that patients with radioresistant tumours determined by the 31-gene signature had worse OS in the radiotherapy-treated group only [34]. There were two studies that used the 31-gene signature in combination with programmed death ligand 1 (PD-L1) status (determined by *CD274* expression) to classify tumours as PD-L1 high/radioresistant versus other in glioma (n = 511; n = 302 radiotherapy, n = 209 no radiotherapy) [21] and glioblastoma (n = 399; n = 284 radiotherapy, n = 115 no radiotherapy) [28] patients from the TCGA. Patients with PD-L1 high/radioresistant tumours had worse OS only in the radiotherapy-treated groups. The final study combined the 31-gene signature and RSI to create a new 12-gene signature containing 11 genes from the 31-gene signature and one gene from RSI [37]. The 31-gene signature and RSI were prognostic in both radiotherapy-treated and non-radiotherapy-treated patients from the TCGA (n = 647) and Chinese Glioma Genome Atlas (n = 741) cohorts.

#### 3.1.3. Interferon-Related DNA Damage Resistance Signature

The interferon-related DNA damage resistance signature (IRDS) was identified in the original literature search. This is a 7-gene signature that classifies a tumour as IRDS+ (DNA damage resistant) or IRDS− (DNA damage sensitive) [40]. Signature genes were identified in vitro and selected for their association with radiotherapy or chemotherapy resistance. A meta-analysis of multiple cohorts of breast cancer patients (n = 1573) showed a significantly higher importance score for the IRDS signature in the prediction of loco-regional recurrence in radiotherapy-treated versus non-radiotherapy-treated patients.

### 3.2. Radiosensitivity Signatures Developed In Vivo

Fourteen radiosensitivity signatures were developed largely using in vivo data (13 purely in vivo, 1 incorporated in vitro data into gene selection). Two studies (prostate cancer [15] and breast cancer [26]) were notable, as they included the retrospective validation of a clinical-grade assay using FFPE tissue in multiple cohorts.

#### 3.2.1. Post-Operative Radiation Therapy Outcomes Score (Decipher PORTOS)

The 24-gene post-operative radiation therapy outcomes score (Decipher PORTOS) [15] was developed in training (n = 196) and validation (n = 330) cohorts formed from five published studies of prostate adenocarcinoma patients treated with radical prostatectomy with or without post-operative radiotherapy. Exact matching (1:1) was performed between patients treated with or without post-operative radiotherapy using a Gleason score, prostate-specific antigen (PSA) concentration, surgical margin, extracapsular extension, seminal vesicle involvement, lymph node involvement and treatment with androgen deprivation therapy. Gene expression data were generated using a microarray analysis of RNA extracted from FFPE tissue in a Clinical Laboratory Improvement Amendments (CLIA)-certified clinical laboratory.

A gene list (n = 1800) was curated from gene ontology and gene set enrichment analysis related to DNA damage response and radiation. A Cox proportional hazards model was used in the training cohort to select 24 genes associated with radiotherapy benefit using distant metastasis as the clinical endpoint. The model was locked before application in the validation cohort. In both cohorts, patients with PORTOS-high tumours had a lower incidence of distant metastasis at 10 years when treated with radiotherapy, whereas an incidence of distant metastasis did not differ between patients treated with or without radiotherapy in those with PORTOS-low tumours.

#### 3.2.2. Adjuvant Radiotherapy Intensification Classifier (ARTIC)

The adjuvant radiotherapy intensification classifier (ARTIC) [26] is a 27-gene signature developed in three publicly available cohorts of breast cancer patients and validated in the phase III SweBCG91-RT trial (n = 748), which randomised women who underwent breast conserving surgery (BCS) with negative margins for stage I–II, node-negative breast cancer between post-operative radiotherapy or no radiotherapy.

Genes were selected for their association with radiotherapy response (based on an endpoint of local recurrence) in one of the training cohorts (n = 343) and then refined to a linear model using a 27-gene signature and age in the remaining cohorts (n = 228, n = 106). The model was locked before external validation in SweBCG91-RT.

In SweBCG91-RT, patients with low ARTIC scores gained a greater benefit from radiotherapy (10-year local recurrence rate 6% with radiotherapy versus 21% without radiotherapy, *p* < 0.001) than those with high ARTIC scoffers (10-year local recurrence rate 25% with radiotherapy versus 32% without radiotherapy), *p* = 0.23). This study did not show a predictive effect for any of the eight other published signatures including MammaPrint, Oncotype Dx and RSI.

#### 3.2.3. Other In Vivo Derived Radiosensitivity Signatures

Seven studies used TCGA data only to generate signatures for soft tissue sarcoma (STS) (n = 2) [17,18], gastric cancer (n = 1) [19], HNSCC (n = 2) [20,33], breast cancer (n = 1) [24] and cervix cancer (n = 1) [35]; four of these used a cross-validated adaptive signature design (CVASD) approach to derive and validate the signature within the same dataset, two split the TCGA cohort into training and validation and one used the TCGA to validate a signature derived in a very small independent cohort. The remaining five studies used multiple publicly available microarray/RNA-Seq cohorts to derive signatures for breast cancer (n = 4) [14,25,32,36] and glioma/glioblastoma (n = 1) [39]. None of these have been externally validated.

### 3.3. Breast Cancer Prognostic Signatures

The literature search revealed four studies assessing the ability of existing commercially available prognostic breast cancer signatures to predict the radiotherapy benefit (MammaPrint [42], Oncotype DCIS [16], Oncotype Dx [23] and Prosigna PAM-50 [31]) and two studies that aimed to derive and validate new breast cancer signatures prognostic for local recurrence [Danish breast cancer cooperative group radiotherapy profile (DBCG-RT) [43] and the profile for the omission of local adjuvant radiation (POLAR) [38]].

#### 3.3.1. MammaPrint

MammaPrint is a 70-gene signature developed to predict the risk of distant recurrence in localised breast cancer [51,52]. There is high-level evidence for its use in guiding decision-making regarding the benefit of adjuvant chemotherapy (EORTC 10041/BIG 3-04 MINDACT) [53] and it is recommended for this purpose in international clinical guidelines [54]. In the original studies, the signature was measured using microarray with fresh-frozen or FFPE tissue; however, this has now been successfully transferred to an RNA-Seq platform for FFPE tissue [55].

A study of MammaPrint was identified via the original literature search. This included patients with T1–3 N0–1 M0 breast cancer treated with BCS or mastectomy with or without post-operative radiotherapy (n = 1053). MammaPrint was an independent prognostic factor for loco-regional recurrence risk (LRR). In patients treated with mastectomy without radiotherapy (n = 501), the 10-year LRR was 13.2% versus 5.8% (*p* = 0.002) in high-risk versus low-risk patients, respectively. There was no statistically significant difference in 10-year LRR in patients treated with mastectomy plus post-operative radiotherapy [12.9% versus 9.2% (*p* = 0.302) in high-risk versus low-risk patients]. MammaPrint may identify patients at a risk of local as well as distant recurrence who are more likely to benefit from post-operative radiotherapy.

#### 3.3.2. Danish Breast Cancer Cooperative Group Radiotherapy Profile (DBCG-RT)

DBCG-RT is a 7-gene signature developed in the DBCG82bc cohort of patients with high-risk breast cancer treated with mastectomy, axillary dissection, and randomised to post-mastectomy radiotherapy (PMRT) or no radiotherapy [43]. RNA was extracted from fresh-frozen tissue and whole transcriptome data generated using microarray analysis in a training cohort (n = 191). Genes were selected for their interaction with PMRT on association with LRR through a Cox proportional hazard model. The signature and algorithm were transferred to a quantitative real-time polymerase chain reaction (qRT-PCR)-based assay for FFPE using samples within the training cohort with a matched FFPE sample (n = 146). This was then validated in 112 patients.

In the training cohort, the 20-year LRR rates were 57% without PMRT and 12% with PMRT (*p* < 0.0001) in the high-risk group (n = 143) and 8% without PMRT versus 9% with PMRT (n = 48) (*p* = 0.93) in the low-risk group. A similar effect was observed in the training cohort, although smaller numbers of patients were analysed (high-risk n = 90, low-risk n = 22).

#### 3.3.3. Oncotype DCIS and Oncotype Dx

Oncotype Dx is a 21-gene signature (16 breast cancer genes and five endogenous control genes used for normalisation) initially developed to predict distance recurrence in node-negative, tamoxifen-treated breast cancer [56]. It is a clinical-grade qRT-PCR-based assay for FFPE tissue that is recommended for use in routine clinical practice internationally [54,57] to guide decision-making regarding systemic therapy for early stage breast cancer patients based on high-level evidence [9,58]. Oncotype DCIS is an abbreviated version of the original Oncotype Dx assay with 12 genes (seven breast cancer genes and five endogenous control genes) that can predict the risk of recurrence after BCS without radiotherapy for ductal carcinoma in situ (DCIS) [59]. It is not currently recommended in clinical guidelines [60].

Oncotype DCIS was evaluated in a large Canadian DCIS cohort (n = 1260) in which patients were treated with BCS alone (n = 571) or BCS plus radiotherapy (n = 689) [16]. Patients were classified based on the Oncotype DCIS score as low-, intermediate-, or high-risk. There were fewer low-risk patients in the radiotherapy-treated cohort (62% no radiotherapy versus 48.1%). A propensity score-adjusted multivariable model was used to identify significant factors associated with local recurrence. Patients deemed high-risk had a greater benefit from radiotherapy [10-year LRR 32.7% no radiotherapy versus 20% radiotherapy in high-risk group; 10-year LRR 16% no radiotherapy versus 9.4% radiotherapy in low-risk group].

Oncotype Dx was assessed in real world data from two large cohorts of patients with T1–2 N1 invasive breast cancer treated with or without PMRT; the National Cancer Database (NCDB) (n = 7332) and the Surveillance, Epidemiology, and End Results (SEER) Program (n = 3087). In both cohorts, there was an overall survival advantage with PMRT for patients classified as low-risk (NCDB—improved OS with PMRT HR 1.70 1.30–2.22 *p* < 0.001; SEER—improved OS with PMRT HR 1.85 CI 1.33–2.57 *p* < 0.001), which was not seen in intermediate-risk (NCDB HR 1.89 CI 0.69–1.14 *p* = 0.35; SEER HR 0.84 CI 0.62–1.14 *p* = 0.26) or high-risk patients (NCDB HR 1.85 CI 1.33–2.57 *p* < 0.001; SEER HR 0.79 CI 0.50–1.23 *p* = 0.28).

#### 3.3.4. Prosigna PAM-50

The Prosigna PAM-50 gene signature is based on expression levels of 50 genes used to classify breast cancers into one of the four breast cancer intrinsic subtypes (Luminal A, Luminal B, HER2-enriched or Basal-like) originally described in 2000 [61]. This was simplified to a 46-gene risk of recurrence (ROR) model measured using the NanoString nCounter platform with FFPE-derived RNA [62].

Prosigna PAM-50 was prognostic for the local recurrence in post-menopausal ER-positive, HER2-negative breast cancer patients (n = 1204) treated with BCS and randomised between different adjuvant endocrine therapies in the ABCSG-8 trial. Whilst most patients (n = 1034) were treated with BCS and post-operative radiotherapy, a small group (n = 170) did not receive adjuvant radiation. In 122/170 patients deemed low-risk by Prosigna PAM-50, the 10-year local recurrence risk was relatively low (5%). However, the hazard ratio for local recurrence in low-risk patients treated with or without radiation was similar to the high-risk patients (Fine–Gray subhazard interaction model *p* = 0.383) indicating a benefit for post-operative radiotherapy for all patients.

#### 3.3.5. Profile for the Omission of Local Adjuvant Radiation (POLAR)

POLAR is a 16-gene signature developed in estrogen receptor (ER)-positive, human epidermal growth factor receptor 2-negative, and node-negative invasive breast cancer treated within two randomised trials of BCS plus or minus post-operative radiotherapy, SweBCG91-RT (n = 597) and Princess Margaret (n = 132). Whole transcriptome data were generated via a microarray analysis of FFPE (SweBCG91-RT) or fresh-frozen (Princess Margaret) tissue in a CLIA certified laboratory. The SweBCG91-RT cohort was split into training (n = 243) and validation (n = 354) and genes that were prognostic for LRR in patients not treated with radiotherapy (n = 131) were selected. These were filtered for being in the most enriched pathways using gene set enrichment analysis and a 16-gene model with LRR as the endpoint was generated using an elastic net regression model. The model and cut-offs were locked before applying to the training cohorts.

In the SweBCG91-RT validation cohort, the 10-year LRR was 5% with radiotherapy and 6% without (*p* = 0.81) for POLAR low-risk patients, whereas there was a benefit for radiotherapy in POLAR high-risk patients (10-year LRR 8% radiotherapy versus 19% no radiotherapy, *p* = 0.0055). This was further validated in the Princess Margaret cohort [10-year LRR 13% radiotherapy versus 7% no radiotherapy POLAR low-risk (*p* = 0.74); 8% radiotherapy versus 22% no radiotherapy (*p* = 0.038) POLAR high-risk].

## 4. Discussion

**Figure 2:**
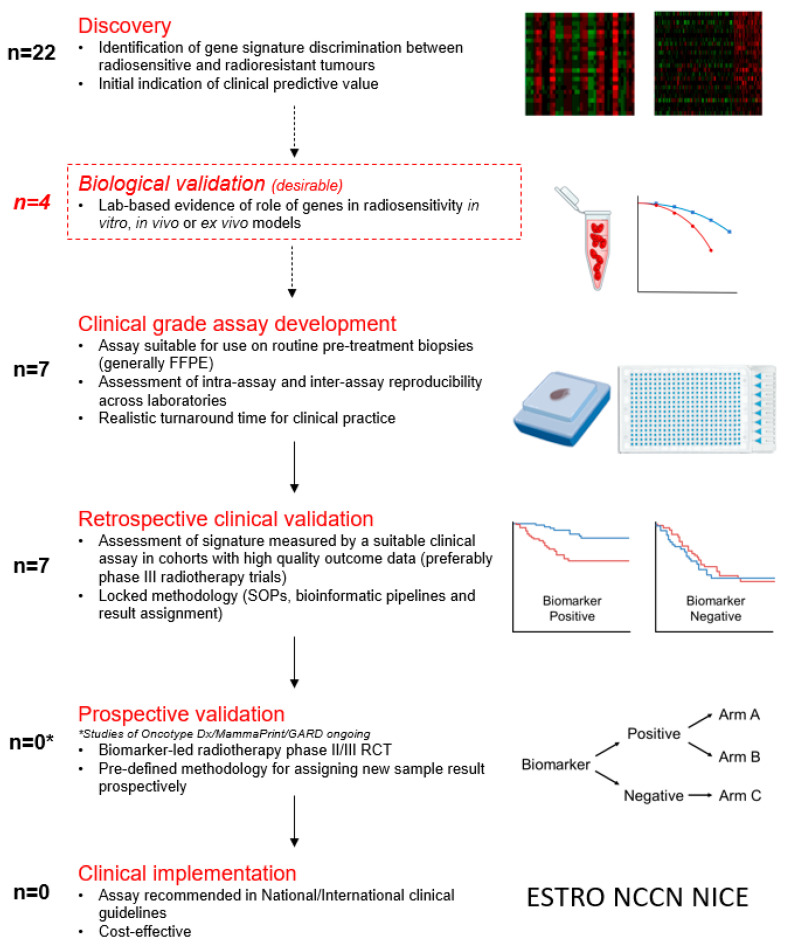
Proposed steps towards clinical implementation of a radiosensitivity biomarker/numbers of identified signatures with evidence of each stage of development are shown. Prospective validation is defined as completion of this step; however, it is noted that this is ongoing or planned for some signatures. FFPE = formalin-fixed, paraffin-embedded; SOP = standard operating procedure; RCT = randomised controlled trial.

There has been a considerable increase in the number of studies evaluating gene signatures to predict radiosensitivity or benefit from radiotherapy over the last decade. An additional 17 signatures were recognised from studies completed since the publication of our first review covering up to 2015. Despite this, none are used in routine clinical practice. Figure 2 proposes a series of steps towards the clinical implementation of a gene signature for the prediction of radiotherapy benefit. It details the numbers of signatures in the literature with evidence of having achieved each step. The barriers to clinical implementation are explored below.

Seventeen signatures were ‘radiosensitivity’ signatures (three pre-2015 and fourteen post-2015) and five were breast cancer signatures prognostic for local recurrence (two pre-2015 and three post-2015). Of the additional 14 radiosensitivity signatures, all were derived using in vivo data and trained on clinical outcomes. These were mostly previously published, publicly available cohorts. It is notable that only one of these signatures has undergone any in vitro biological validation of the genes included. Seven studies only used TCGA data and four of these used a cross-validated adaptive signature design (CVASD) to derive and validate the signature within the same cohort in three cancer types. Two of the CVASD studies [TCGA soft tissue sarcoma (STS) cohort] were published by the same authors in the same year and derived two different signatures with only a single overlapping gene (Table 4). In addition, the quality of the clinical baseline and outcome data is often questionable or incomplete. Studies relying entirely on these cohorts without generating new cohorts with more detailed participant data can be limited. For example, there are four studies of breast cancer signatures that use overall survival or progression free/recurrence free survival as endpoints when breast radiotherapy intervention has a larger impact on improving local control when compared with overall survival [63]. Information regarding confounding factors is also often missing, such as the STS TCGA cohort, a disease site in which grade is an important prognostic factor [64] but is not available for inclusion in multi-variate analyses. While these resources are an excellent starting point for biomarker discovery and supporting validation, the lack of further development of these signatures indicates that they are not a substitute for proper external and prospective validation.

Of the in vivo derived signatures, only two (ARTIC and Decipher PORTOS) had a high quality, with a retrospective validation of a clinical-grade assay with a locked methodology before application to the validation cohorts. This is important as it has been shown that variation in methodology, such as normalisation methods for transcriptomic data, can dramatically affect the results and performance of radiosensitivity signatures [65]. Currently, Decipher PORTOS is not recommended for routine clinic use in the ASCO guidelines due to a lack of prospective validation in a controlled trial. An assessment in ongoing prospective clinical trials such as RTOG-9601 (NCT00002874) is planned, although these are not specifically biomarker-led trials [66].

The radiosensitivity signatures aim to measure a specific aspect of tumour biology, namely intrinsic radiosensitivity. It has been previously demonstrated that signatures > 100 genes in length in which genes are selected at random are likely to be prognostic in breast cancer due to general transcriptional upregulation in more aggressive cancers [67]. Genes selected mainly based on their association with clinical outcomes are therefore more likely to be simply prognostic, rather than specifically a measure of radiosensitivity. The general paucity of the biological validation of the identified signatures may explain the lack of any significant overlap in signature genes (Table 4). There were only 37 overlapping genes (35 in two signatures, 2 in four signatures). CCNB1 encodes cyclin B1 which is involved in the progression of the cell cycle from the G2 to M phase. MMP11 encodes a matrix metalloproteinase and as such is involved in the enzymatic breakdown of the extracellular matrix. These two genes were included in four overlapping gene expression signatures, and both are involved in processes manipulated by cancers during disease development. Both genes are represented in the Oncotype Dx and Prosigna PAM-50 signatures which were developed initially as predictors for breast cancer recurrence. The presence of these genes may represent predictive factors of recurrence in breast and other cancers more so than specific predictors of radiosensitivity. One gene was in two signatures published by the same authors in the same dataset, ten were between breast cancer prognostic signatures and only five genes from in vitro derived signatures appeared in another signature derived in vivo. This contrasts with radiotherapy signatures developed to measure tumour hypoxia as a determinant of radioresistance, where the top 20 overlapping genes appeared in between 12 and 31 of 32 published signatures [68]. This is likely due to more emphasis on the biological validation step, as it is easier to model hypoxia in the laboratory or compare to other hypoxia markers in vivo. Future directions may include the use of better models to determine gene function, for example, the use of clustered regularly interspaced short palindromic repeat (CRISPR) [69] genome editing to knock out or upregulate potential radiosensitivity genes to facilitate appropriate gene selection.

Two of the four radiosensitivity signatures (RSI and the 31-gene signature) developed in vitro have undergone more comprehensive clinical evaluation, having been assessed in multiple cohorts by independent groups. Despite being trained to predict in vitro SF_2_, both have been criticised for their ability to reflect this, showing poor performance compared to randomly selected genes in multiple cell lines. RadSigBench is proposed as a comprehensive benchmarking framework that could be used in the biological validation of future signatures [70]. An independent re-analysis of the original derivation dataset found that RSI performed worse than random guessing in predicting in vitro SF_2_ [71].

The 31-gene signature derived by Kim et al. has been assessed in HNSCC in a single study and more extensively in primary brain tumours (glioma grade 2–3 and WHO grade IV glioblastoma) alone and in combination with immune status [PD-L1 (*CD274* RNA expression)]. There is no clinical-grade assay for application and these studies lack a consistent methodology for sample classification. The first validation study uses the original 31-gene signature in the TCGA glioma cohort. There are two further studies in the TCGA glioma (lower grade) and glioblastoma cohorts combining the 31-gene (or in one study 30-genes) with the PD-L1 status to identify a radioresistant/PD-L1 high group. Finally, one study uses an abbreviated version of the signature plus one gene from RSI in the TCGA and CGGA cohorts. A further retrospective validation with locked bioinformatic pipelines and signature result assignment methods is needed to progress this signature any further, preferably in a new cohort with better clinical annotation.

RSI is the most extensively studied radiosensitivity signature with respect to clinical application. The search criteria identified six studies in four tumour sites inclusive of 2707 patients. A recent review by the group that developed RSI/GARD reported that this has been validated in 12 disease sites (21 cohorts) in >4000 patients [72]. Some of these studies were excluded from this review due to low patient numbers (e.g., lung n = 95 [73], melanoma n = 42 [74], penile n = 10 [75], pancreas n = 73 [76], rectal n = 14 [47], oesophagus n = 12 [47]). In other excluded studies, RSI was assessed only in cohorts in which all patients received radiotherapy, such as HNSCC n = 235 [77] or the phase III BCON bladder cancer cohort n = 151 (RSI showed a non-significant association with local relapse free survival on univariate analysis) [78]. More extensive retrospective validation would be helpful in these disease sites. RSI was assessed in addition to the reported signature in three studies and its predictive value was not validated [25,26,32].

The most studied tumour site with regard to RSI is breast (~30% of patients in this review); therefore, the authors have taken the signature forward for prospective validation in this site first. RSI/GARD has been commercialised through Cvergenx, a spin-out company of the Moffit Cancer Centre [79]. GARD is currently undergoing prospective validation in a biomarker-led phase II randomised trial [Genomically Guided Radiation Therapy in the Management of Triple Negative Breast Cancer (NCT05528133)] with completion anticipated in 2026–2027 [80]. It should be noted that the retrospective validation and prospective trial consider patients treated with conventionally (50 Gy in 25 fractions) or moderately hypofractionated radiotherapy (42.56 Gy in 16 fractions). Clinical practice in breast cancer is moving towards using ultra-hypofractionated whole breast schedules (26 Gy in 5 fractions) and the use of simultaneous integrated boost doses so it will be important to ensure that GARD remains applicable to these regimens [81].

The breast cancer prognostic signatures are used to identify patients with less aggressive tumours and a lower local recurrence risk who stand to benefit less from adjuvant radiotherapy. These signatures are not for specifically measuring radiosensitivity; they were originally developed to predict distance recurrence risk and therefore select patients at a high-risk of harbouring micro-metastatic disease with more potential to benefit from chemotherapy. It has also been proposed more recently that they may identify tumours that are more sensitive to chemotherapy [82]. They are further ahead in development than radiosensitivity signatures, as the existing ones already have technically validated, clinical-grade, commercially available assays.

The study of Prosigna PAM-50 demonstrated that local recurrence rates were low in low-risk patients. The signature did not predict response to radiation, as there was still a benefit in the low-risk population. However, as the local recurrence rates in the low-risk group may be acceptable to some patients, the test could help guide discussions regarding the risks and benefits of post-operative radiation. The prospective evaluation of the omission of post-operative radiotherapy for Prosigna PAM-50 low-risk patients is ongoing in the single arm, phase II PRECISION trial (NCT02653733) [83].

It is interesting that in DCIS, there was increased benefit for adjuvant radiotherapy in high-risk patients by Oncotype DCIS, whereas in invasive breast cancer the Oncotype Dx low-risk patients gained benefit from radiotherapy (with an endpoint of overall survival) compared to intermediate-risk and high-risk patients. It was suggested by the authors that this could be due to more significance of a local recurrence in low-risk patients with less likelihood of developing distant metastases. Early results (at 5 years) of the single-arm IDEA study, in which post-operative radiotherapy was omitted for Oncotype Dx low-risk, ER-positive, HER2-negative breast cancer patients are encouraging, with low rates of ipsilateral breast recurrence (3.3% age 50–59, 3.6% age 60–69) reported at five years [84]. This will be further assessed in two non-inferiority, randomised, phase III studies, CCTG MA.39 TAILOR RT (NCT03488693) [85] and NRG-BR007/DEBRA (NCT04852887) (which also allows the use of MammaPrint) [86].

Once there are established biomarkers of radiosensitivity, more individualised radiotherapy plans will be possible. The radiotherapy technique and dose can be personalised to achieve optimal treatment outcomes and a reduction in side effects. Comparative trials of different treatment regimens for patients stratified by a radiosensitivity biomarker will be required to confirm the clinical benefit and safety of such an approach.

## 5. Conclusions and Future Directions

In conclusion, there has been an explosion in newly discovered radiosensitivity signatures over the last decade, largely due to publicly available cohorts with whole transcriptome gene expression data such as TCGA, which are powerful resources for biomarker discovery. However, the quality of signatures that are heavily reliant on the bioinformatic analysis of publicly available data (which are often incomplete) with little or no biological or external validation is poor. This is evident in the fact that none of the signatures discovered this way appear to have the ability to transition to prospective biomarker-led trials or clinical implementation. Of the reported radiosensitivity signatures (excluding prognostic breast cancer signatures), RSI/GARD is the most extensively studied by some way and the results of the prospective phase II trial are anticipated with great interest.

To establish biologically guided radiotherapy in the clinic, future work should focus on (1) developing clinically relevant models such as well characterised patient-derived cell lines and organoids to study radiosensitivity mechanisms and involved genes in the laboratory and benchmarking compared to other signatures; (2) building biobanks alongside large radiotherapy randomised controlled trials with dose variance (to demonstrate an interaction between radiosensitivity signature and dose) in a range of tumour types as a resource for signature validation; (3) developing robust and cost-effective assays for use in routine pre-treatment biopsies that can be delivered in a timely manner within current healthcare systems; and (4) integrating with biomarkers of other determinants of radiation response, such as tumour hypoxia.

## Figures and Tables

**Table 1:** Literature search exclusion criteria.

Not original research article (reviews, letters, comments)
Not radiotherapy biomarkers (irrelevant, diagnostic markers, prognostic markers, chemotherapy benefit, immunotherapy benefit, normal tissue toxicity)
Pre-clinical or mechanistic studies of radiosensitivity
Assessed in a small number of patients (<100)
Not assessed as predictive marker (no non-radiotherapy-treated control cohort)
Predicting benefit of addition of another modality to radiotherapy (hypoxia modification, concurrent chemotherapy)

**Table 2:** Studies of gene signatures predictive of radiotherapy benefit.

Signature	Derivation	Proposed Biological Mechanism	Tumour Type	Cohort	*N*	Cohort Type	Prognosis/Prediction	Endpoint	Assay/Tissue	Reference
10-gene signature (RSI)	in vitro	Radiosensitivity	Prostate	Moffitt	618	Validation	Prediction	Distant metastasis	Microarray/FF	Torres-Roca et al., 2014 [13].
3-gene signature	in vivo	Cell adhesion molecules	TNBC	Shanghai	32	Training	-	-	Microarray/FF	Wushou et al., 2015 [14].
				Shanghai	166	Validation	Prediction	RFS		
24-gene signature (PORTOS)	in vivo	DNA damage repair and radiation response	Prostate	Mayo Clinic	196	Training	Prediction	Distant metastasis	Microarray/FFPE	Zhao et al., 2016 [15].
				Pooled	330	Validation	Prediction	Distant metastasis		
7-gene signature (Oncotype Dx DCIS)	in vitro	Proliferation	DCIS	Ontario DCIS	1260	Validation	Prediction	LRR	qRT-PCR/FFPE	Rakovitch et al., 2017 [16].
26-gene signature	in vivo	Radiosensitivity	STS	TCGA	253	Training/Validation	Prediction	OS	RNA-Seq/FF	Tang et al., 2017 [17].
65-gene signature	in vivo	Radiosensitivity	STS	TCGA	218	Training/Validation	Prediction	OS	RNA-Seq/FF	Tang et al., 2017 [18].
11-gene signature	in vivo	Radiosensitivity	Gastric	TCGA	371	Training/Validation	Prediction	OS	RNA-Seq/FF	Zhou et al., 2017 [19].
5-miRNA signature	in vivo	Not stated	HNSCC	TCGA	553	Training	-	-	miRNA-Seq/FF	Chen et al., 2018 [20].
				TCGA	154	Training	Prognosis	OS		
				TCGA	153	Validation	Prognosis	OS		
				TCGA	509	Validation	Prediction	OS		
31-gene signature + PD-L1	in vivo	Cell junction and adhesion	Glioma	TCGA	511	Validation	Prediction	OS	RNA-Seq/FF	Jang et al., 2018 [21].
10-gene signature (RSI)	in vitro	Radiosensitivity	Breast	Sweden	307	Validation	Prediction	LR	Nanostring/FF	Sjöström et al., 2018 [22].
16-gene signature (Oncotype Dx) *	in vitro	Prognostic signature	Breast	NCDB	7332	Validation	Prediction	OS	RT-PCR/FFPE	Goodman et al., 2018 [23].
				SEER	3087	Validation	Prediction	OS		
30-gene signature	in vivo	Radiosensitivity	Breast	TCGA	700	Training/Validation	Prediction	OS	RNA-Seq/FF	Ji et al., 2018 [24].
34-gene signature	in vivo	Radiosensitivity	Breast	GSE30682	343	Training	Prognosis	LRFS	Microarray/FF	Cui et al., 2018 [25].
				NKI	319	Validation	Prognosis	RFS		
				GSE2034	286	Validation	Prognosis	RFS		
				METABRIC	262	Validation	Prediction	DSS		
27-gene signature (ARTIC)	in vivo	Not stated	Breast	GSE30692	343	Training	Prognosis	LR	Microarray/FF	Sjöström et al., 2019 [26].
				NKI	228	Training	Prognosis	LR	Microarray/FF	
				GSE103746	106	Training	Prognosis	LR	Microarray/FF	
				SweBCG91-RT	748	Validation	Prediction	LRR	Microarray/FFPE	
10-gene signature (RSI)	in vitro	Radiosensitivity	Endometrial	Moffitt	204	Validation	Prediction	Pelvic control	Microarray/FFPE	Mohammadi et al., 2020 [27].
30-gene signature	in vitro	Cell junction and adhesion	GBM	TCGA	399	Validation	Prediction	OS	RNA-Seq/FF	Jang et al., 2020 [28].
10-gene signature (RSI)	in vitro	Radiosensitivity	Prostate	Manchester	386	Validation	Prediction	PFS	Microarray/FFPE	Thiruthaneeswaran et al., 2020 [29].
10-gene signature (RSI)	in vitro	Radiosensitivity	PDAC	TCGA	182	Training	Prognosis	OS	RNA-Seq/FF	Nishiwada et al., 2021 [30].
				ICGC	94	Validation	Prognosis	OS		
				Nara	145	Training	Prognosis	OS	RT-PCR/FFPE	
				Kumamoto	112	Validation	Prognosis	OS		
				pre-NACRT cohort	56	Validation	Prediction	Pathological response		
46-gene signature (PAM-50)	in vivo	Prognostic signature	Breast	ABCSG-8	1204	Validation	Prediction	LR	Nanostring/FF	Fitzal et al., 2021 [31].
4-gene signature	in vivo	Transcriptional regulation	Breast	TCGA	976	Training	Prediction	OS	RNA-Seq/FF	Yan et al., 2021 [32].
				METABRIC	1798	Training/Validation	Prediction	OS	Microarray/FF	
3-gene signature	in vivo	Radiosensitivity/immune status	HNSCC	TCGA	236	Training	Prediction	OS	RNA-Seq/FF	Sun et al., 2021 [33].
				TCGA	156	Validation	Prediction	OS		
31-gene signature	in vitro	Cell junction and adhesion	HNSCC	TCGA	288	Validation	Prediction	OS	RNA-Seq/FF	Dai et al., 2021 [34].
185-gene signature	in vivo	Various	Cervix	TCGA	9	Derivation	-	PFS	RNA-Seq/FFPE	Kim et al., 2022 [35].
				TCGA	273	Validation	Prediction	PFS	RNA-Seq/FF	
11-gene signature	in vivo	Not stated	Breast	TCGA	937	Training	Prediction	PFS	RNA-Seq/FF	Shen et al., 2022 [36].
				E-TABM-158	130	Validation	Prediction	PFS		
12-gene signature	in vivo	Radiosensitivity	Glioma	CGGA	748	Validation	Prediction	OS	RNA-Seq/FF	Wu et al., 2022 [37].
				TCGA	647	Validation	Prediction	OS		
16-gene signature (POLAR)	in vivo	Not stated	Breast	SweBCG91-RT	243	Training	Prediction	LRR	Microarray/FFPE	Sjöström et al., 2023 [38].
				SweBCG91-RT	354	Validation	Prediction	LRR		
				Princess Margaret	132	Validation	Prediction	LRR	Microarray/FF	
9-gene signature	in vitro/in vivo	Not stated	Glioma/GBM	GSE7696	84	Derivation	-	-	RNA-Seq/FF	Zhang et al., 2023 [39].
				TCGA -GBM	152	Training/Validation	Prediction	PFS	RNA-Seq/FF	
				TCGA-low grade glioma	616	Training/Validation	Prediction	PFS	RNA-Seq/FF	
				SMU-NFH	31	Validation	Prediction	PFS	RNA-Seq/FFPE	
				CGGA	501	Validation	Prediction	OS	RNA-Seq/FF	

miRNA = micro RNA; PD-L1 = programmed death-ligand 1; RSI = radiosensitivity index; TNBC = triple negative breast cancer; DCIS = ductal carcinoma in situ; STS = soft tissue sarcoma; HNSCC = head and neck squamous carcinoma; GBM = glioblastoma multiforme; PDAC = pancreatic ductal adenocarcinoma; OS = overall survival; LRFS = local recurrence free survival; RFS = recurrence free survival; DSS = disease specific survival; LRR = locoregional recurrence; PFS = progression free survival; TCGA = The Cancer Genome Atlas; NCDB = National Cancer Database; SEER = Surveillance, Epidemiology, and End Results Program; NKI = Netherlands Cancer Institute; METABRIC = Molecular Taxonomy of Breast Cancer International Consortium; ICGC = International Cancer Genome Consortium; CGGA = Chinese Glioma Genome Atlas; NACRT = Neoadjuvant Chemoradiotherapy; FF = fresh-frozen; FFPE = formalin-fixed, paraffin-embedded. * There have been prospective trials using Oncotype Dx (21-gene recurrence score) but not specifically assessing prediction of benefit of radiotherapy.

**Table 3:** Studies of gene signatures predictive of radiotherapy benefit (pre-2015).

Biomarker	Derivation	Proposed Biological Mechanism	Tumour Type	Cohort	n	Cohort Type	Prognosis/Prediction	Endpoint	Clinical Assay	Reference
7-gene signature	in vitro	IFN-related DNA damage resistance	Breast	Multiple (meta-analysis)	1573	Validation	Prediction	LRC	Microarray/FF	Weichselbaum et al., 2008 [40].
10-gene signature (RSI)	in vitro	Radiosensitivity	Breast	Karolinska	159	Validation	Prediction	RFS	Microarray/FF	Eschrich et al., 2012 [41].
				Erasmus	344	Validation	Prediction	MFS		
70-gene signature (Mammaprint) *	in vivo	Prognostic signature	Breast	NKI	1053	Validation	Prediction	LRR	Microarray/FFPE	Drukker et al., 2014 [42].
7-gene signature (DBCG-RT)	in vivo	Not stated	Breast	DBCG82bc	191	Training	Prediction	LRR	Microarray/FF	Tramm et al., 2014 [43].
				DBCG82bc	112	Validation	Prediction	LRR	qRT-PCR/FFPE	
31-gene signature	in vitro	Radiosensitivity	Glioma	GSE16011	263	Validation	Prediction	OS	RNA-Seq/FF	Meng et al., 2014 [44].
				TCGA	463	Validation	Prognosis	OS	Microarray/FF	

RSI = radiosensitivity index; OS = overall survival; LRC = loco-regional control; RFS = recurrence free survival; MFS = metastasis free survival; LRR = locoregional recurrence; OS = overall survival; NKI = Netherlands Cancer Institute; DBCG = Danish Breast Cancer Cooperative Group; TCGA = The Cancer Genome Atlas. * There have been prospective trials using Mammaprint but not specifically assessing prediction of benefit of radiotherapy.

**Table 4:** Genes common to identified gene signatures.

Gene	Signatures (n)	Function	References
*ACTN1*	2	F-actin cross-linking protein which is thought to anchor actin to a variety of intracellular structures. This is a bundling protein.	Meng et al., 2014 [44], Kim et al., 2022 [35].
*ANLN*	2	Required for cytokinesis.	Zhao et al., 2016 [15], Fitzal et al., 2021 (Prosigna PAM-50) [31].
*BAG1*	2	Acts as a nucleotide-exchange factor promoting the release of ADP from the HSP70 and HSC70 proteins thereby triggering client/substrate protein release.	Goodman et al., 2018 (Oncotype Dx) [23], Fitzal et al., 2021 (Prosigna PAM-50) [31].
*BCL2*	2	Regulates cell death by controlling the mitochondrial membrane permeability. Appears to function in a feedback loop system with caspases.	Goodman et al., 2018 (Oncotype Dx) [23], Fitzal et al., 2021 (Prosigna PAM-50) [31].
*BTG3*	2	Overexpression impairs serum-induced cell cycle progression from the G0/G1 to S phase.	Cui et al., 2018 [25], Sjöström et al., 2019 [26].
*CCNB1*	4	Essential for the control of the cell cycle at the G2/M (mitosis) transition.	Goodman et al., 2018 (Oncotype Dx) [23], Cui et al., 2018 [25], Sjöström et al., 2019 [26], Fitzal et al., 2021 (Prosigna PAM-50) [31].
*CDC5L* *	2	DNA-binding protein involved in cell cycle control. May act as a transcription activator. Plays a role in pre-mRNA splicing as core component of precatalytic, catalytic and post-catalytic spliceosomal complexes.	Tang et al., 2017 [17], Tang et al., 2017 [18].
*CENPF*	2	Required for kinetochore function and chromosome segregation in mitosis.	Sjöström et al., 2019 [26], Fitzal et al., 2021 (Prosigna PAM-50) [31].
*CKB*	2	Reversibly catalyses the transfer of phosphate between ATP and various phosphogens.	Fitzal et al., 2021 (Prosigna PAM-50) [31], Shen et al., 2022 [36].
*CLGN*	2	Functions during spermatogenesis as a chaperone for a range of client proteins that are important for sperm adhesion onto the egg zona pellucida and for subsequent penetration of the zona pellucida.	Cui et al., 2018 [25], Sun et al., 2021 [33].
*DAG1*	2	The dystroglycan complex is involved in a number of processes including laminin and basement membrane assembly, sarcolemmal stability, cell survival, peripheral nerve myelination, nodal structure, cell migration, and epithelial polarisation.	Meng et al., 2014 [44], Ji et al., 2018 [24].
*DRAM1*	2	Lysosomal modulator of autophagy that plays a central role in p53/TP53-mediated apoptosis.	Zhao et al., 2016 [15], Tang et al., 2017 [17]
*DTL*	2	Substrate-specific adapter of a DCX (DDB1-CUL4-X-box) E3 ubiquitin-protein ligase complex required for cell cycle control, DNA damage response and translesion DNA synthesis.	Drukker et al., 2014 (MammaPrint) [42], Zhao et al., 2016 [15].
*ERBB2*	2	Protein tyrosine kinase that is part of several cell surface receptor complexes.	Goodman et al., 2018 (Oncotype Dx) [23], Fitzal et al., 2021 (Prosigna PAM-50) [31].
*ESR1*	2	Nuclear hormone receptor.	Goodman et al., 2018 (Oncotype Dx) [23], Fitzal et al., 2021 (Prosigna PAM-50) [31].
*GNG11*	2	G-protein transmembrane signalling.	Zhao et al., 2016 [15], Sjöström et al., 2023 (POLAR) [38].
*HCLS1*	2	Substrate of the antigen receptor-coupled tyrosine kinase. Plays a role in antigen receptor signalling for both clonal expansion and deletion in lymphoid cells. May also be involved in the regulation of gene expression.	Meng et al., 2014 [44], Zhao et al., 2016 [15].
*KNTC2*	2	Acts as a component of the essential kinetochore-associated NDC80 complex, which is required for chromosome segregation and spindle checkpoint activity.	Drukker et al., 2014 (MammaPrint) [42], Fitzal et al., 2021 (Prosigna PAM-50) [31].
*KPNA2*	2	Functions in nuclear protein import as an adapter protein for nuclear receptor KPNB1.	Cui et al., 2018 [25], Sjöström et al., 2023 (POLAR) [38].
*KRT14*	2	The nonhelical tail domain is involved in promoting KRT5-KRT14 filaments to self-organise into large bundles.	Zhao et al., 2016 [15], Fitzal et al., 2021 (Prosigna PAM-50) [31].
*KRT15*	2	The keratins are intermediate filament proteins responsible for the structural integrity of epithelial cells and are subdivided into cytokeratins and hair keratins.	Sun et al., 2021 [33], Kim et al., 2022 [35].
*MDM2*	2	E3 ubiquitin-protein ligase that mediates ubiquitination of p53/TP53, leading to its degradation by the proteasome.	Cui et al., 2018 [25], Fitzal et al., 2021 (Prosigna PAM-50) [31].
*MELK*	2	Serine/threonine-protein kinase involved in various processes such as cell cycle regulation, self-renewal of stem cells, apoptosis and splicing regulation.	Drukker et al., 2014 (MammaPrint) [42], Fitzal et al., 2021 (Prosigna PAM-50) [31].
*MKI67*	2	Required to maintain individual mitotic chromosomes dispersed in the cytoplasm following nuclear envelope disassembly	Goodman et al., 2018 (Oncotype Dx) [23], Fitzal et al., 2021 (Prosigna PAM-50) [31].
*MMD*	2	Involved in the dynamics of lysosomal membranes associated with microglial activation following brain lesion.	Cui et al., 2018 [25], Kim et al., 2022 [35].
*MMP11*	4	May play an important role in the progression of epithelial malignancies.	Goodman et al., 2018 (Oncotype Dx) [23], Fitzal et al., 2021 (Prosigna PAM-50) [31], Kim et al., 2022 [35], Sjöström et al., 2023 (POLAR) [38].
*MORF4L2*	2	Component of the NuA4 histone acetyltransferase complex which is involved in transcriptional activation of select genes principally by acetylation of nucleosomal histone H4 and H2A.	Kim et al., 2022 [35], Shen et al., 2022 [36]
*MX1*	2	Interferon-induced dynamin-like GTPase with antiviral activity against a wide range of RNA viruses and some DNA viruses.	Weichselbaum et al., 2008 [40], Kim et al., 2022 [35].
*NAT1*	2	Participates in the detoxification of a plethora of hydrazine and arylamine drugs.	Tang et al., 2017 [17], Fitzal et al., 2021 (Prosigna PAM-50) [31].
*ORC6L*	2	Component of the origin recognition complex (ORC) that binds origins of replication.	Drukker et al., 2014 (MammaPrint) [42], Fitzal et al., 2021 (Prosigna PAM-50) [31].
*PGR*	2	The steroid hormones and their receptors are involved in the regulation of eukaryotic gene expression and affect cellular proliferation and differentiation in target tissues.	Goodman et al., 2018 (Oncotype Dx) [23], Fitzal et al., 2021 (Prosigna PAM-50) [31].
*PLK2*	2	Tumour suppressor serine/threonine-protein kinase involved in synaptic plasticity, centriole duplication and G1/S phase transition.	Zhao et al., 2016 [15], Zhang et al., 2023 [39].
*POSTN*	2	Induces cell attachment and spreading and plays a role in cell adhesion.	Kim et al., 2022 [35], Zhang et al., 2023 [39].
*PYGB*	2	Glycogen phosphorylase that regulates glycogen mobilisation.	Meng et al., 2014 [44], Cui et al., 2018 [25].
*RGS4*	2	Inhibits signal transduction by increasing the GTPase activity of G protein alpha subunits thereby driving them into their inactive GDP-bound form.	Tang et al., 2017 [17], Kim et al., 2022 [35].
*SCUBE2*	2	SHH long-range signalling by binding to the dually lipid-modified SHH (ShhNp) and by promoting ShhNp mobilisation, solubilisation and release from the cell membrane.	Drukker et al., 2014 (MammaPrint) [42], Goodman et al., 2018 (Oncotype Dx) [23].
*STAT1*	2	Signal transducer and transcription activator that mediates cellular responses to interferons (IFNs), cytokine KITLG/SCF and other cytokines and other growth factors	Weichselbaum et al., 2008 [40], Eschrich et al., 2012 [41].

* Duplicate in two signatures published by the same group derived in the same dataset.
